# Parental Optimism Improves Youth Psychological Well-Being: Family Cohesion and Youth Optimism as Serial Mediators

**DOI:** 10.3390/healthcare10101832

**Published:** 2022-09-22

**Authors:** Wei Qi, Jing Shi, Lijuan Cui

**Affiliations:** 1 Institute of Brain and Education Innovation, East China Normal University, Shanghai 200062, China; 2 School of Educational Science, Shenyang Normal University, Shenyang 110034, China; 3 School of Psychology and Cognitive Science, East China Normal University, Shanghai 200062, China

**Keywords:** optimism, family cohesion, psychological well-being

## Abstract

Focusing on the family system, this study simultaneously examined the effects of the parental factor, family functioning, and individual factor on youth psychological well-being. Overall, 332 youths and their parents were involved in this research and responded to an online questionnaire measuring parental optimism, family cohesion, youth optimism, and youth psychological well-being. The results suggested that (1) parental optimism was positively related to youth psychological well-being; (2) both family cohesion and youth optimism mediated the connection between parental optimism and youth psychological well-being; and (3) the link between parental optimism and youth psychological well-being was mediated by family cohesion and youth optimism in sequence. The present study reveals the underlying mechanism of how to improve youth psychological well-being from within the family system.

## 1. Introduction

An individual’s psychological well-being is a crucial sign of their quality of life [1]. Ryff and Keyes [2] assert that psychological well-being (PWB) is derived from emotional experiences as well as from realizing one’s potential. As positive psychology has grown in popularity, more and more studies have focused on the positive effects of PWB on individuals and explored both the general and situation-specific factors that influence individual PWB in different cultural and social contexts [3,4]. Early adulthood is an important period for individuals to develop psychological maturity and well-being; therefore, uncovering factors and developmental pathways that influence well-being in early adulthood and finding ways to improve well-being are of great importance to promoting desirable physical and psychological development in youths [5]. To date, limited studies have looked at how the family system influences youth psychological development [6], and studies that explore the combined influences of the family system and individual factors are even fewer. To fill the research gap, this study aims to explore the underlying mechanisms that can improve the PWB of young adults by considering the parental factor, family functioning, and individual factor simultaneously.

### 1.1. Parental Optimism and Youth PWB

As the earliest site of human physical and psychological development, the family is an important environment for individuals to grow up happy and healthy. A surging amount of research underlines the value of the family to the PWB of youths [7]. Numerous studies have demonstrated that parent-related factors, as important components of the family system, directly affect how young people develop PWB [8,9,10]. However, prior research has primarily looked into the effect of parenting styles on children’s psychological health, relatively ignoring the significance of some personality traits, i.e., optimism, that parents themselves possess. Optimism is an individual’s disposition to anticipate favorable events in his or her life [11]. Researchers have discovered that more optimistic parents are more satisfied with their children [12]. Higher parental optimism levels predict more positive and less negative feelings among parents [13]. Mothers’ and fathers’ optimism leads them to adopt appropriate parenting practices, hence enhancing peer competence in their children [14]. Furthermore, optimism can also alleviate parenting stress by allowing parents to show more positive expectations about their children’s future weight, education level, and financial income [15]. These findings suggest that parental optimism, as an important personality trait in parents, is prone to positively influence young people’s PWB.

### 1.2. Parental Optimism, Family Cohesion, and Youth PWB

On one hand, positive parental factors may elicit favorable family functioning. Previous research has indicated that positive parental factors, such as appropriate parental behaviors and parenting style, usually result in high-quality, caring, and supportive family functioning [16]. Family cohesion is the extent to which members of the family are devoted to and supportive of one another [17], which is a crucial indicator of family functioning. According to the family resilience process model, the influence of parents on their offspring may be impacted by the specific functioning of the family system [18]. Thus, parental optimism as a positive parental factor also has the potential to influence family functioning, such as family cohesion. On the other hand, previous research has indicated that family functioning is one of the important predictors of parents’ and children’s life satisfaction [19] and PWB [20]. Previous studies have also provided relevant evidence on the possible mediating role of family cohesion. For example, in Lorenzo-Blanco et al.’s [21] six-wave longitudinal study, the relationship between parent cultural stress and youth emotional well-being was significantly mediated by family functioning. Thus, family cohesion as the manifestation of family functioning can be considered a mediator between parental optimism and young people’s PWB.

### 1.3. Parental Optimism, Youth Optimism, and Youth PWB

In the family, parents’ words and actions often have a subtle influence on their children. Social learning theory suggests that within the family, young people see their parents as role models and thus imitate many of their behaviors and attitudes [22]. Similarly, social contagion theory (SCT) posits that people transmit and display the same feelings and behaviors because of social contagion [23]. The transmission of psychological conditions (e.g., happiness) has been documented between siblings [24], among peers [25], and in classrooms [26]. Moreover, several cross-sectional studies have provided evidence for an observed transfer of PWB from parents to children [27,28]. It can be seen that young people can observe and learn strong beliefs and perseverance in the face of difficulties from highly optimistic parents, and the positive attitudes of parents are passed on to young people, who then internalize their parents’ optimism as a personality trait of their own. Based on the above theory, optimistic parents are likely to foster optimistic young people. Furthermore, optimism has proven to be a significant indicator of PWB [29,30]. Thus, parental optimism may affect youth optimism, which in turn impacts youth PWB.

### 1.4. Parental Optimism, Family Cohesion, Youth Optimism, and PWB

Although the above literature suggests that parental optimism, family cohesion, and youth optimism may all play a vital role in youth PWB, the mechanisms among these factors are not yet clarified. Person–context interaction theory suggests that environmental factors can be divided into distal and proximal ones depending on the distance to the individual’s experience [31]. Researchers have further pointed out that the distal environment often influences the individual’s development by directly affecting the function of the proximal environment [32]. According to this theory, when exploring the influence young people receive from their parents within the family system, parental characteristics (parental optimism) can be seen as the distal environment, which influences the proximal environment (youth optimism) through family functioning (family cohesion) and ultimately has an effect on youth PWB.

### 1.5. The Present Study

**Figure 1:**
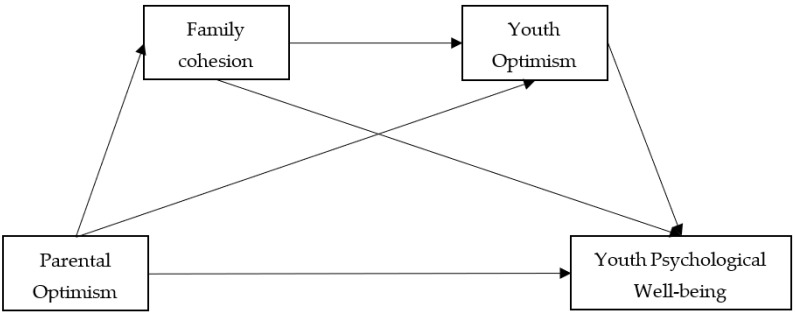
Hypothetical model.

This study explores the underlying mechanics that influence young people’s PWB based on the family system. Specifically, the present study considers the predictive effects of the parental factor (parental optimism), family functioning (family cohesion), and individual factor (youth optimism) on emerging adults’ well-being (youth PWB) simultaneously and proposes a serial mediation model (see Figure 1) to examine the following four hypotheses:

**Hypothesis** **1**. *Parental optimism is positively associated with youth PWB*.

**Hypothesis** **2**. *Family cohesion may serve as a mediator between parental optimism and youth PWB*.

**Hypothesis** **3**. *Youth optimism may mediate the association between parental optimism and youth PWB*.

**Hypothesis** **4**. *The association between parental optimism and youth PWB is mediated by family cohesion and youth optimism in sequence*.

## 2. Materials and Methods

### 2.1. Participants and Procedure

Young adults aged 18 to 25 years old and their parents were recruited through the online survey platform Questionnaire Star to participate in this study. The youths and their parents clicked on different questionnaire links to fill in the corresponding online questionnaires, and both questionnaires were matched by the same invitation code. A total of 411 pairs of questionnaires were collected from the youths and their parents. After excluding missing information (if participants did not complete the questionnaire) and those who did not answer the questionnaire carefully, 332 valid pairs of questionnaires were received in total. Among the parent participants, 127 were fathers, and 205 were mothers; M_age_ = 48.62 years, and SD_age_ = 5.35 years. Among the youth participants, 149 were males, and 183 were females; M_age_ = 22.16 years, and SD_age_ = 1.44 years.

### 2.2. Measures

#### 2.2.1. Parental and Youth Optimism

The Life Orientation Test-Revised (LOT-R) [33] was employed to evaluate dispositional optimism in parents and youths separately. In addition to four filter items, three items on the LOT-R assess pessimism, and three items estimate optimism. The LOT-R is rated on a 5-point Likert scale, with 1 representing strong disagreement and 5 representing strong agreement. Typical items include: “In uncertain times, I usually expect the best”. After reversing the coding for the pessimism items, the scores for the optimism and pessimism dimensions were added together to generate a total score. A higher total score indicated stronger optimism. The Cronbach’s α values for parental optimism and youth optimism were 0.804 and 0.781, respectively, in this study.

#### 2.2.2. Family Cohesion

Youths used the cohesion subscale of the Family Adaptability and Cohesion Evaluation Scales IV [34] to evaluate family cohesion. The subscale for family cohesion comprises seven items. Participants provided answers on a 5-point Likert scale, where 1 = does not describe our family and 5 = describes our family very well. An example item is: “Family members feel very close to each other”. Higher scores indicate greater family cohesion. The Cronbach’s α for this scale was 0.863.

#### 2.2.3. PWB

Youth PWB was assessed by a shortened version of the Scale of PWB [35]. The scale incorporates 39 items and measures six dimensions related to PWB: self-acceptance (6 items, an example item being “I like most aspects of my personality”), positive relations with others (6 items, an example item being “People would describe me as a giving person, willing to share my time with others”), environmental mastery (6 items, an example item being “In general, I feel I am in charge of the situation in which I live”), purpose in life (6 items, an example item being “Some people wander aimlessly through life, but I am not one of them”), personal growth (7 items, an example item being “I think it is important to have new experiences that challenge how you think about yourself and the world”), and autonomy (8 items, an example item being “I have confidence in my opinions, even if they are contrary to the general consensus”). A 6-point Likert scale is used to rate the scale, with 1 representing total disagreement and 6 representing total agreement. A higher score indicates a higher degree of PWB. The scale’s Cronbach’s α was 0.832.

### 2.3. Data Analysis

IBM SPSS Statistics 24.0 and Model 6 of the PROCESS macro [36] were applied for data analysis. Statistical estimations for common method bias, descriptive statistics, correlations, and serial mediation were conducted to examine data quality and Hypotheses 1–4.

## 3. Results

### 3.1. Common Method Bias (CMB) Analysis

This study examined CMB via exploratory factor analysis for Harman’s single factor test [37]. According to the results, 10 factors have eigenvalues above 1, and the first component accounted for 18.25% of the variance, below the threshold of 40%. This result indicates that although the present study used a questionnaire method for data collection, no significant CMB was seen.

### 3.2. Descriptive Analysis

Descriptive statistics and Pearson correlation were evaluated for parental optimism, family cohesion, youth optimism, and youth PWB (see Table 1). The correlation analysis showed that youth PWB and parental optimism were positively correlated (*r* = 0.27, *p* < 0.001), which supported Hypothesis 1. In addition, significant relationships were found between main variables, which provided a basis for the examination of the serial mediation model of central interest.

### 3.3. Serial Mediation Model Test

**Figure 2:**
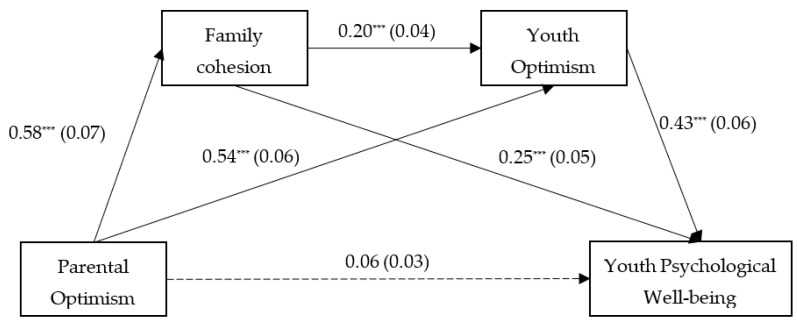
Serial mediation model of family cohesion and youth optimism between parental optimism and youth PWB. Dashed lines represent nonsignificant paths. *** *p* < 0.001.

Model 6 from the PROCESS macro [36] was deployed to analyze the serial mediation model of family cohesion and youth optimism between parental optimism and youth PWB. Table 2 and Figure 2 illustrate the results of the regression analysis. Taking youth age and gender as control variables, parental optimism positively predicted family cohesion (*β* = 0.58, *SE* = 0.07, *p* < 0.001) as well as youth optimism (*β* = 0.54, *SE* = 0.06, *p* < 0.001); family cohesion positively predicted youth optimism (*β* = 0.20, *SE* = 0.04, *p* < 0.001). When parental optimism, family cohesion, and youth optimism simultaneously predicted youth PWB, both family cohesion (*β* = 0.25, *SE* = 0.05, *p* < 0.001) and youth optimism (*β* = 0.43, *SE* = 0.06, *p* < 0.001) turned out to be significantly positive indicators of youth PWB, while parental optimism no longer significantly predicted youth PWB (*β* = 0. 06, *SE* = 0.03, *p* = 0. 219).

Subsequently, this study was evaluated with a 95% confidence interval (CI) by conducting bootstrapping procedures with 5000 bootstrapped samples to examine mediation effects in the proposed model, with youth age and gender as control variables. Family cohesion and youth optimism fully mediated the association between parental optimism and youth PWB. Three pathways contributed to the total mediation effect: the mediation effect of parental optimism on youth PWB through family cohesion was 0.14, 95% CI = [0.07, 0.16]; the mediation effect of parental optimism on youth PWB through youth optimism was 0.23, 95% CI = [0.15, 0.29]; and the serial mediation effect of parental optimism on youth PWB through family cohesion and youth optimism in sequence was 0.05, 95% CI = [0.02, 0.06]. The bootstrap 95% CI for the above three mediating pathways did not contain 0, indicating that all three mediation effects reached a significant level (see Table 3). Thus, Hypotheses 2–4 were supported.

## 4. Discussion

### 4.1. The Association between Parental Optimism and Youth PWB

The current study suggests that parental optimism is strongly and positively connected with youth PWB. Although youths gradually leave their families to focus on their development during early adulthood, parental personality traits remain important environmental factors influencing youth development [38,39]. Highly optimistic parents adopt positive coping styles in the face of difficulties within or outside the family [12]. Moreover, parents’ optimistic tendencies act as a model for young people, so youths growing up with such parents also learn to adopt positive attitudes in adversities and thus have higher levels of well-being.

### 4.2. Mediation Effects of Family Cohesion and Youth Optimism

According to our findings, family cohesion mediates the association between parental optimism and youth PWB. The positive link between family cohesion and parental optimism found in this study is consistent with previous research [40]. One possible explanation is that parents who display high levels of optimism often hold bright outlooks for youths’ futures and are more inclined to support and encourage them, which leads to a mutually supportive and caring family functioning [41]. Young people who grow up in families with such high levels of cohesion thus achieve higher levels of PWB. Thus, a highly cohesive family atmosphere provided by optimistic parents can be an important psychological resource for youths in their development, supporting youths in their pursuit of life’s values and thus enhancing youth PWB.

Furthermore, the current study demonstrates that young people’s optimism serves as a mediator between parental optimism and youth PWB. It is worth noting that youth optimism exhibits the largest effect size among the three mediating pathways in the present study, which suggests youth optimism is a noteworthy mediator for the effect of parental optimism on youth PWB. Such results theoretically support the applicability of SCT in family systems [23]. As a positive psychological construct, optimism is characterized by taking a hopeful attitude toward the future [42]. Highly optimistic parents do not see difficulties or setbacks as insurmountable, but rather accept their current situation and respond positively to it [43]. At the same time, highly optimistic parents not only have positive expectations of themselves but also tend to see their children in a positive light [44]. According to SCT, parents’ optimistic tendencies could be transmitted to young people. Highly optimistic parents adopt more positive behaviors and fewer maladaptive behaviors in parent–child interactions, which contributes to higher levels of youth optimism. Moreover, youths’ growth is significantly influenced by optimism, which becomes a key source of their happiness [45]. Prior studies have indicated that more optimistic individuals are happier, healthier, and less likely to have physical, academic, psychological, and behavioral problems than those with low levels of optimism [46,47]. Therefore, the mediating impact of youth optimism discovered in this study is consistent with earlier research. As this study first investigated the mediation effect of young people’s optimism on the parental optimism and youth PWB relationship, our findings extend the knowledge on the link between optimism and well-being based on the SCT. Specifically, this study highlights parental optimism as an important resource in the family system that can be passed on to young people and foster an optimistic personality trait in youths, which enhances their PWB.

More importantly, this study found that family cohesion and youth optimism acted as serial mediators between parental optimism and youth PWB. Previous research has found that optimistic individuals exhibit more tolerance and patience during interpersonal interaction [48] and give higher interpersonal trust to others [49], both of which contribute to closer interpersonal relationships. The present study suggests that the same phenomena are found in families, with highly optimistic parents also cultivating high levels of family cohesion. Furthermore, the present study indicates that family cohesion could contribute to youth PWB through youth optimism. Such findings reveal the process of how parents influence youth well-being by taking into account the parental factor, family functioning, and individual factor simultaneously, which expands the perspective of previous research. Specifically, the serial mediation effect (parental optimism→family cohesion→youth optimism→youth PWB) revealed by this study expands upon prior studies which mostly considered the influence of parental, family, or individual factors on children separately [50]. Therefore, the serial mediation model in the present study provides new insights into how parents, families, and young people themselves jointly influence youth PWB.

### 4.3. Limitations

Although the present study reveals the underlying mechanism by which the parental factor, family functioning, and individual factor combine to influence youth PWB, there are still some limitations. First, as this is the first study to investigate youth PWB by systematically considering factors within the family, the generalizability of our findings remains unknown. To evaluate the study’s generalizability, it would be beneficial for future research to boost the size of the current sample and replicate this study in other countries. Secondly, this study’s cross-sectional design greatly hindered its ability to establish causal inferences between factors. For future research, a longitudinal design could be used to discover causal evidence for the serial mediation model. Third, the parental optimism data used in the present study did not distinguish between fathers and mothers, and future research could explore whether fathers’ optimism and mothers’ optimism have different effects on youth PWB.

## 5. Conclusions

From a family system perspective, this study adds to the existing body of literature by examining a serial mediation model that provides a comprehensive explanation of how the parental factor, family functioning, and individual factor jointly improve youth PWB. The findings suggest that parental optimism is positively correlated with youth PWB. In addition, both family cohesion and youth optimism can be single mediators in the connection between parental optimism and youth PWB. More importantly, the link between parental optimism and youth PWB also can be mediated by family cohesion and youth optimism in sequence. These findings uncover the significance of the family system in the development of youth PWB. In particular, this study highlights the effects of positive parental factors and good family functioning in boosting individual psychological development that endures into early adulthood, which has important implications for interventions to improve youth PWB.

## Figures and Tables

**Table 1:** Descriptive analysis and correlations of main variables (N = 332).

	M	SD	1	2	3	4
1. Parental optimism	3.36	0.71	1			
2. Family cohesion	3.09	0.88	0.25 ***	1		
3. Youth optimism	3.47	0.69	0.32 ***	0.58 ***	1	
4. Youth PWB	4.12	0.84	0.27 ***	0.61 ***	0.73 ***	1

*Note.* *** *p* < 0.001.

**Table 2:** Regression analysis of the variables in the serial mediation model.

Outcome Variables	Predictive Variables	*R^2^*	*F*	*β*	*SE*	*t*
Family cohesion	Parental optimism	0.18	54.91 ***	0.58	0.07	14.87 ***
Youth optimism	Parental optimism	0.12	26.57 ***	0.54	0.06	9.32 ***
	Family cohesion			0.20	0.04	4.69 ***
Youth psychological wellbeing	Parental optimism	0.47	114.06 ***	0.06	0.03	1.18
	Family cohesion			0.25	0.05	10.03 ***
	Youth optimism			0.43	0.06	12.18 ***

*Note.* The variables are standardized before being entered into the regression equation. *** *p* < 0.001.

**Table 3:** Analysis of the mediation effects in the serial mediation model.

	Effect	SE	95% CI
Direct Effect	0.06	0.03	[−0.02, 0.08]
Parental optimism→family cohesion→youth PWB	0.14	0.02	[0.07, 0.16]
Parental optimism→youth optimism→youth PWB	0.23	0.04	[0.15, 0.29]
Parental optimism→family cohesion→youth optimism→youth PWB	0.05	0.01	[0.02, 0.06]
Total mediation effect	0.42	0.04	[0.33, 0.50]
Total effect	0.48	0.04	[0.41, 0.62]

## Data Availability

The data presented in this study are available from the corresponding author on reasonable request.
